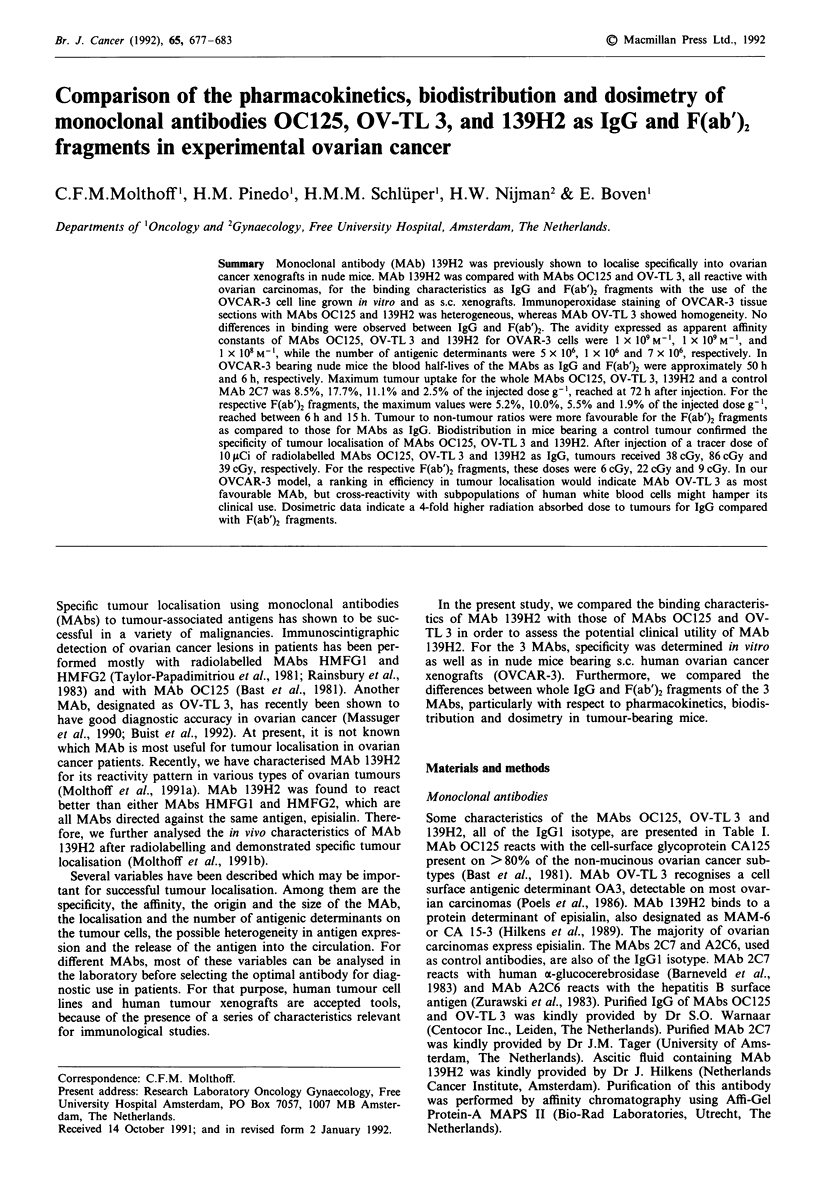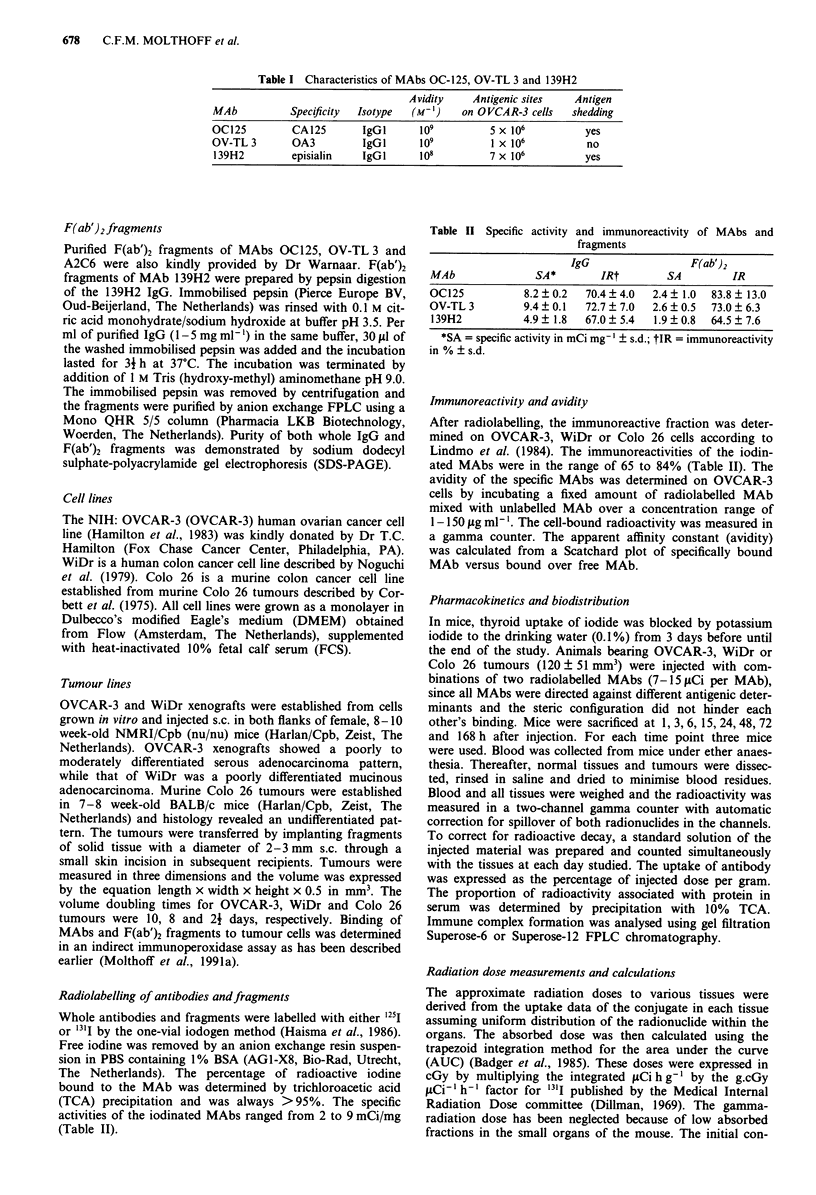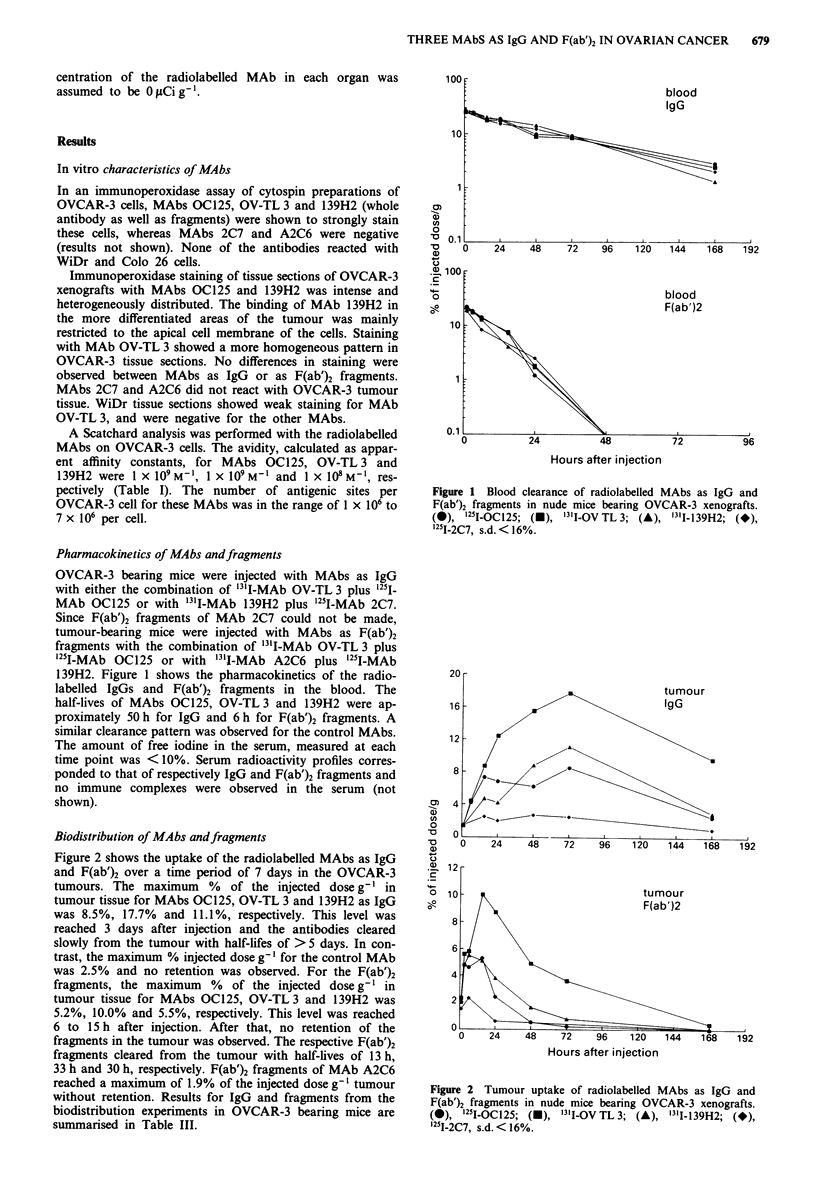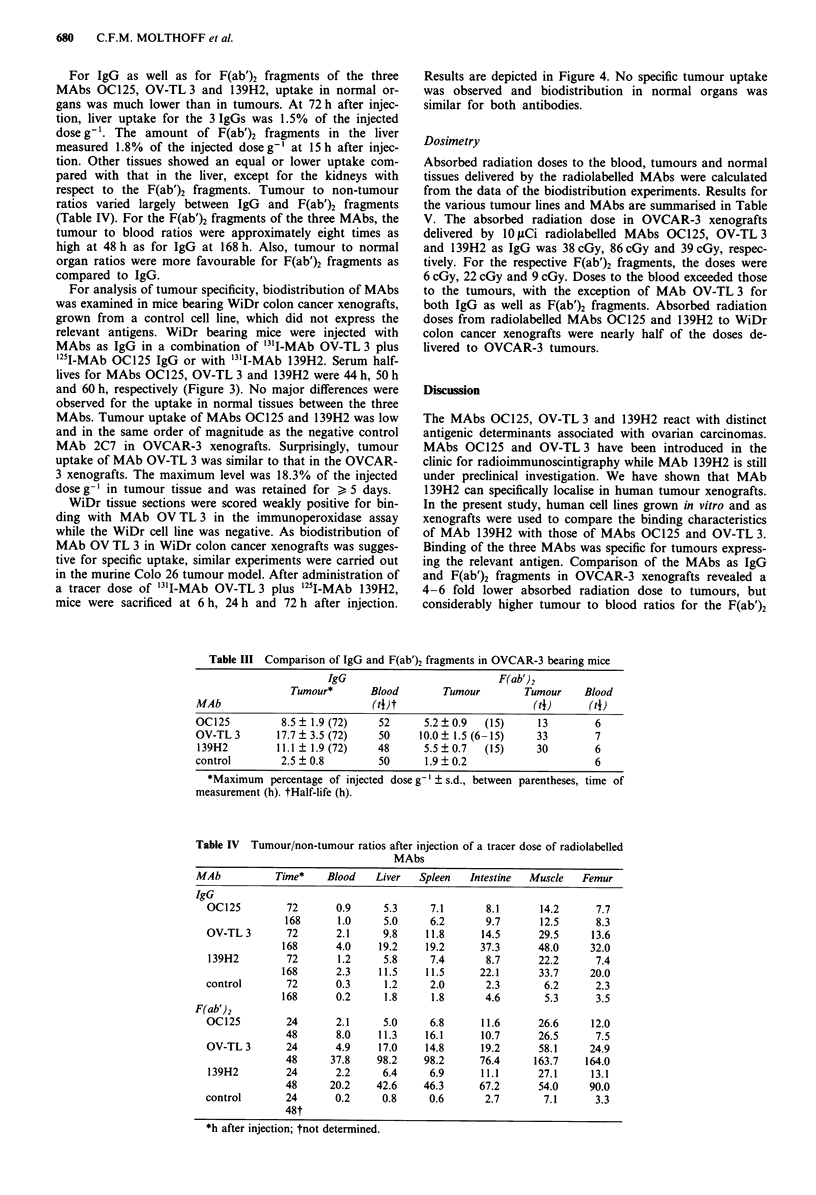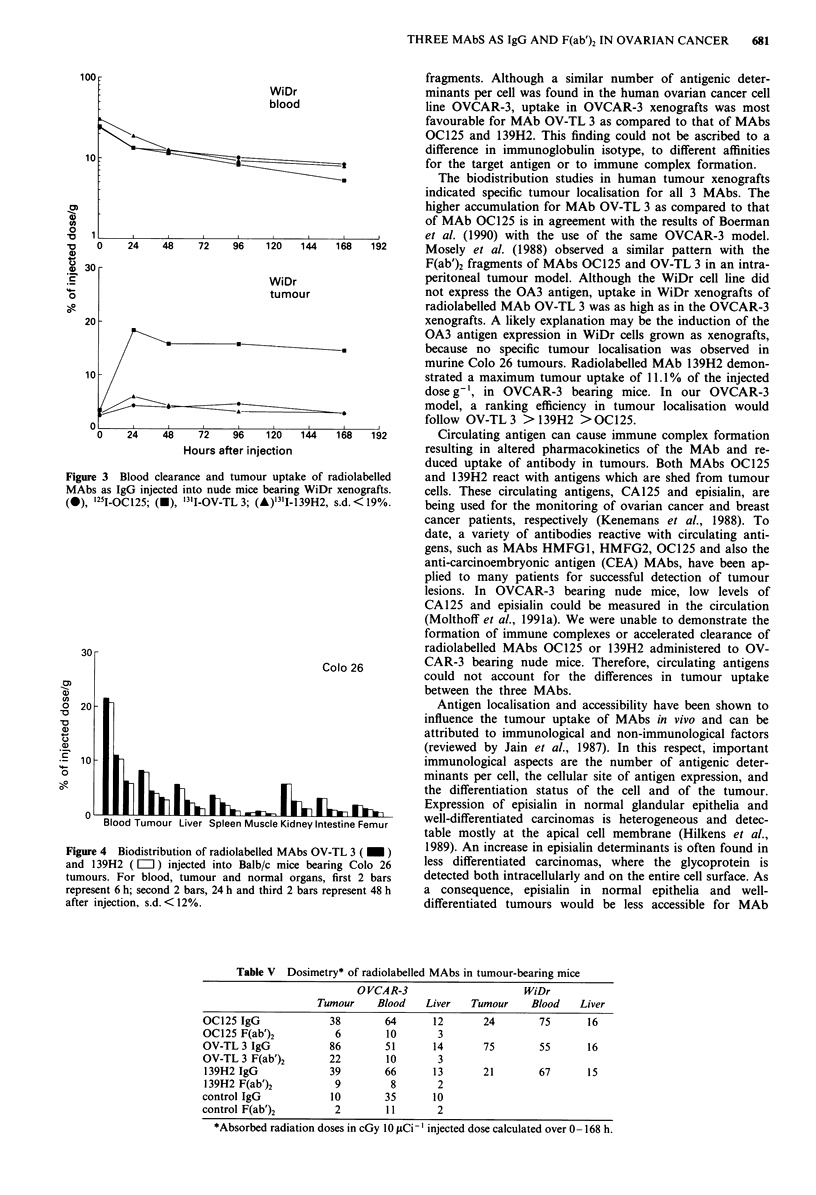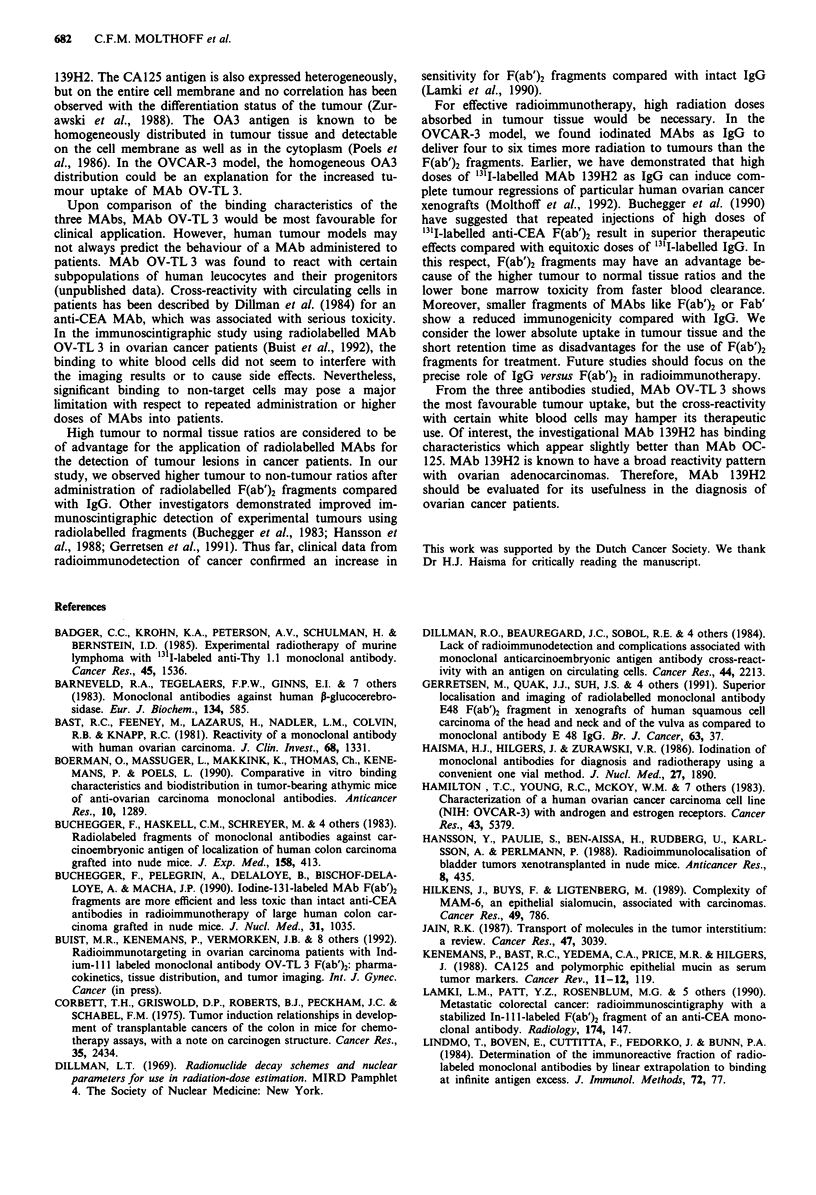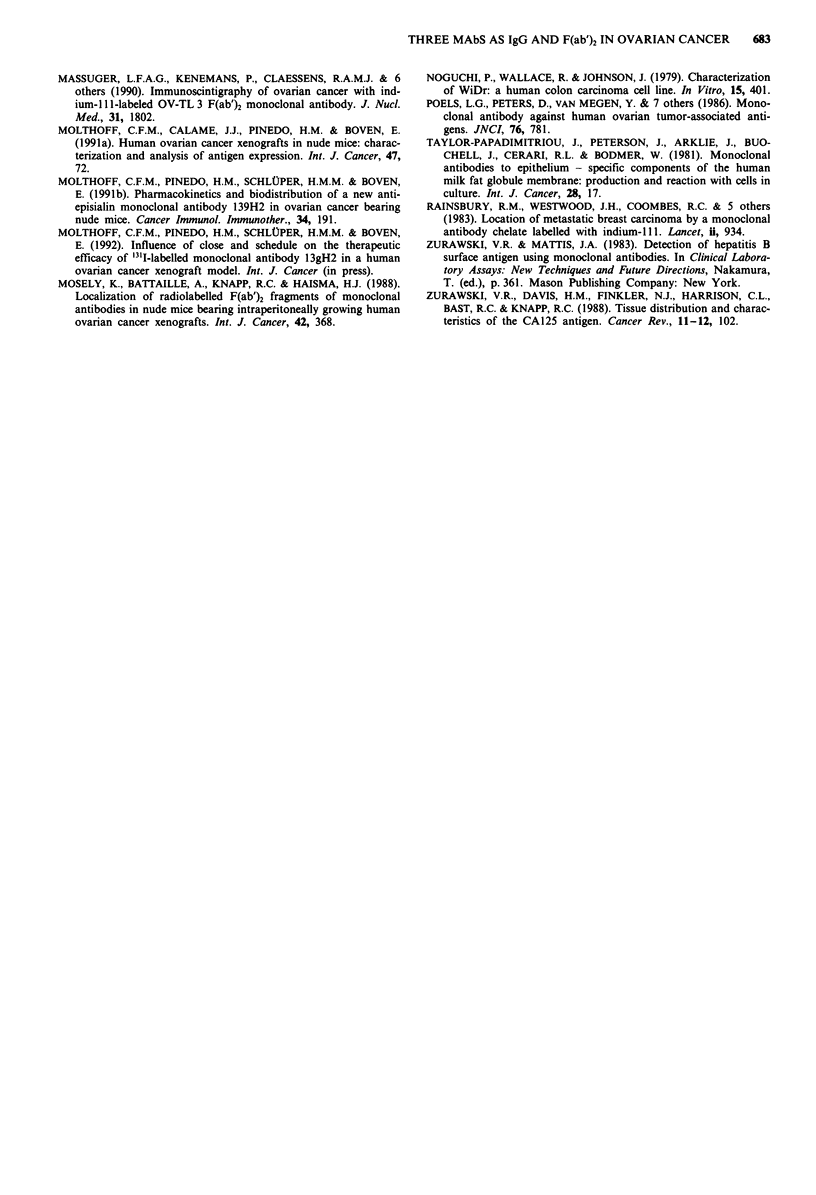# Comparison of the pharmacokinetics, biodistribution and dosimetry of monoclonal antibodies OC125, OV-TL 3, and 139H2 as IgG and F(ab')2 fragments in experimental ovarian cancer.

**DOI:** 10.1038/bjc.1992.144

**Published:** 1992-05

**Authors:** C. F. Molthoff, H. M. Pinedo, H. M. Schlüper, H. W. Nijman, E. Boven

**Affiliations:** Department of Oncology, Free University Hospital, Amsterdam, The Netherlands.

## Abstract

Monoclonal antibody (MAb) 139H2 was previously shown to localise specifically into ovarian cancer xenografts in nude mice. MAb 139H2 was compared with MAbs OC125 and OV-TL 3, all reactive with ovarian carcinomas, for the binding characteristics as IgG and F(ab')2 fragments with the use of the OVCAR-3 cell line grown in vitro and as s.c. xenografts. Immunoperoxidase staining of OVCAR-3 tissue sections with MAbs OC125 and 139H2 was heterogeneous, whereas MAb OV-TL 3 showed homogeneity. No differences in binding were observed between IgG and F(ab')2. The avidity expressed as apparent affinity constants of MAbs OC125, OV-TL 3 and 139H2 for OVAR-3 cells were 1 x 10(9) M-1, 1 x 10(9) M-1, and 1 x 10(8) M-1, while the number of antigenic determinants were 5 x 10(6), 1 x 10(6) and 7 x 10(6), respectively. In OVCAR-3 bearing nude mice the blood half-lives of the MAbs as IgG and F(ab')2 were approximately 50 h and 6 h, respectively. Maximum tumour uptake for the whole MAbs OC125, OV-TL 3, 139H2 and a control MAb 2C7 was 8.5%, 17.7%, 11.1% and 2.5% of the injected dose g-1, reached at 72 h after injection. For the respective F(ab')2 fragments, the maximum values were 5.2%, 10.0%, 5.5% and 1.9% of the injected dose g-1, reached between 6 h and 15 h. Tumour to non-tumour ratios were more favourable for the F(ab')2 fragments as compared to those for MAbs as IgG. Biodistribution in mice bearing a control tumour confirmed the specificity of tumour localisation of MAbs OC125, OV-TL 3 and 139H2. After injection of a tracer dose of 10 microCi of radiolabelled MAbs OC125, OV-TL 3 and 139H2 as IgG, tumours received 38 cGy, and 9 cGy. In our OVCAR-3 model, a ranking in efficiency in tumour localisation would indicate MAb OV-TL 3 as most favourable MAb, but cross-reactivity with subpopulations of human white blood cells might hamper its clinical use. Dosimetric data indicate a 4-fold higher radiation absorbed dose to tumours for IgG compared with F(ab')2 fragments.


Br. J. Cancer (1992), 65, 677-683 © Macmillan Press Ltd., 1992

### Comparison of the pharmacokinetics, biodistribution and dosimetry of monoclonal antibodies OC125, OV-TL 3, and 139H2 as IgG and F(ab')$_2$ fragments in experimental ovarian cancer

C.F.M.Molthoff[1], H.M. Pinedo[1], H.M.M. Schlüper[1], H.W. Nijman[2] & E. Boven[1]

*Departments of [1]Oncology and [2]Gynaecology, Free University Hospital, Amsterdam, The Netherlands.*

**Summary** Monoclonal antibody (MAb) 139H2 was previously shown to localise specifically into ovarian cancer xenografts in nude mice. MAb 139H2 was compared with MAbs OC125 and OV-TL 3, all reactive with ovarian carcinomas, for the binding characteristics as IgG and F(ab')$_2$ fragments with the use of the OVCAR-3 cell line grown *in vitro* and as s.c. xenografts. Immunoperoxidase staining of OVCAR-3 tissue sections with MAbs OC125 and 139H2 was heterogeneous, whereas MAb OV-TL 3 showed homogeneity. No differences in binding were observed between IgG and F(ab')$_2$. The avidity expressed as apparent affinity constants of MAbs OC125, OV-TL 3 and 139H2 for OVAR-3 cells were $1 \times 10^9$ M$^{-1}$, $1 \times 10^9$ M$^{-1}$, and $1 \times 10^8$ M$^{-1}$, while the number of antigenic determinants were $5 \times 10^6$, $1 \times 10^6$ and $7 \times 10^6$, respectively. In OVCAR-3 bearing nude mice the blood half-lives of the MAbs as IgG and F(ab')$_2$ were approximately 50 h and 6 h, respectively. Maximum tumour uptake for the whole MAbs OC125, OV-TL 3, 139H2 and a control MAb 2C7 was 8.5%, 17.7%, 11.1% and 2.5% of the injected dose g$^{-1}$, reached at 72 h after injection. For the respective F(ab')$_2$ fragments, the maximum values were 5.2%, 10.0%, 5.5% and 1.9% of the injected dose g$^{-1}$, reached between 6 h and 15 h. Tumour to non-tumour ratios were more favourable for the F(ab')$_2$ fragments as compared to those for MAbs as IgG. Biodistribution in mice bearing a control tumour confirmed the specificity of tumour localisation of MAbs OC125, OV-TL 3 and 139H2. After injection of a tracer dose of 10 μCi of radiolabelled MAbs OC125, OV-TL 3 and 139H2 as IgG, tumours received 38 cGy, 86 cGy and 39 cGy, respectively. For the respective F(ab')$_2$ fragments, these doses were 6 cGy, 22 cGy and 9 cGy. In our OVCAR-3 model, a ranking in efficiency in tumour localisation would indicate MAb OV-TL 3 as most favourable MAb, but cross-reactivity with subpopulations of human white blood cells might hamper its clinical use. Dosimetric data indicate a 4-fold higher radiation absorbed dose to tumours for IgG compared with F(ab')$_2$ fragments.

Specific tumour localisation using monoclonal antibodies (MAbs) to tumour-associated antigens has shown to be successful in a variety of malignancies. Immunoscintigraphic detection of ovarian cancer lesions in patients has been performed mostly with radiolabelled MAbs HMFG1 and HMFG2 (Taylor-Papadimitriou *et al.*, 1981; Rainsbury *et al.*, 1983) and with MAb OC125 (Bast *et al.*, 1981). Another MAb, designated as OV-TL 3, has recently been shown to have good diagnostic accuracy in ovarian cancer (Massuger *et al.*, 1990; Buist *et al.*, 1992). At present, it is not known which MAb is most useful for tumour localisation in ovarian cancer patients. Recently, we have characterised MAb 139H2 for its reactivity pattern in various types of ovarian tumours (Molthoff *et al.*, 1991a). MAb 139H2 was found to react better than either MAbs HMFG1 and HMFG2, which are all MAbs directed against the same antigen, episialin. Therefore, we further analysed the *in vivo* characteristics of MAb 139H2 after radiolabelling and demonstrated specific tumour localisation (Molthoff *et al.*, 1991b).

Several variables have been described which may be important for successful tumour localisation. Among them are the specificity, the affinity, the origin and the size of the MAb, the localisation and the number of antigenic determinants on the tumour cells, the possible heterogeneity in antigen expression and the release of the antigen into the circulation. For different MAbs, most of these variables can be analysed in the laboratory before selecting the optimal antibody for diagnostic use in patients. For that purpose, human tumour cell lines and human tumour xenografts are accepted tools, because of the presence of a series of characteristics relevant for immunological studies.

Correspondence: C.F.M. Molthoff.
Present address: Research Laboratory Oncology Gynaecology, Free University Hospital Amsterdam, PO Box 7057, 1007 MB Amsterdam, The Netherlands.
Received 14 October 1991; and in revised form 2 January 1992.

In the present study, we compared the binding characteristics of MAb 139H2 with those of MAbs OC125 and OV-TL 3 in order to assess the potential clinical utility of MAb 139H2. For the 3 MAbs, specificity was determined *in vitro* as well as in nude mice bearing s.c. human ovarian cancer xenografts (OVCAR-3). Furthermore, we compared the differences between whole IgG and F(ab')$_2$ fragments of the 3 MAbs, particularly with respect to pharmacokinetics, biodistribution and dosimetry in tumour-bearing mice.

### Materials and methods

### *Monoclonal antibodies*

Some characteristics of the MAbs OC125, OV-TL 3 and 139H2, all of the IgG1 isotype, are presented in Table I. MAb OC125 reacts with the cell-surface glycoprotein CA125 present on >80% of the non-mucinous ovarian cancer subtypes (Bast *et al.*, 1981). MAb OV-TL 3 recognises a cell surface antigenic determinant OA3, detectable on most ovarian carcinomas (Poels *et al.*, 1986). MAb 139H2 binds to a protein determinant of episialin, also designated as MAM-6 or CA 15-3 (Hilkens *et al.*, 1989). The majority of ovarian carcinomas express episialin. The MAbs 2C7 and A2C6, used as control antibodies, are also of the IgG1 isotype. MAb 2C7 reacts with human α-glucocerebrosidase (Barneveld *et al.*, 1983) and MAb A2C6 reacts with the hepatitis B surface antigen (Zurawski *et al.*, 1983). Purified IgG of MAbs OC125 and OV-TL 3 was kindly provided by Dr S.O. Warnaar (Centocor Inc., Leiden, The Netherlands). Purified MAb 2C7 was kindly provided by Dr J.M. Tager (University of Amsterdam, The Netherlands). Ascitic fluid containing MAb 139H2 was kindly provided by Dr J. Hilkens (Netherlands Cancer Institute, Amsterdam). Purification of this antibody was performed by affinity chromatography using Affi-Gel Protein-A MAPS II (Bio-Rad Laboratories, Utrecht, The Netherlands).

**Table I** Characteristics of MAbs OC-125, OV-TL 3 and 139H2

| *MAb* | *Specificity* | *Isotype* | *Avidity* ($M^{-1}$) | *Antigenic sites on OVCAR-3 cells* | *Antigen shedding* |
|---|---|---|---|---|---|
| OC125 | CA125 | IgG1 | $10^9$ | $5 \times 10^6$ | yes |
| OV-TL 3 | OA3 | IgG1 | $10^9$ | $1 \times 10^6$ | no |
| 139H2 | episialin | IgG1 | $10^8$ | $7 \times 10^6$ | yes |

*F(ab')₂ fragments*

Purified $F(ab')_2$ fragments of MAbs OC125, OV-TL 3 and A2C6 were also kindly provided by Dr Warnaar. $F(ab')_2$ fragments of MAb 139H2 were prepared by pepsin digestion of the 139H2 IgG. Immobilised pepsin (Pierce Europe BV, Oud-Beijerland, The Netherlands) was rinsed with 0.1 M citric acid monohydrate/sodium hydroxide at buffer pH 3.5. Per ml of purified IgG (1–5 mg $ml^{-1}$) in the same buffer, 30 µl of the washed immobilised pepsin was added and the incubation lasted for 3½ h at 37°C. The incubation was terminated by addition of 1 M Tris (hydroxy-methyl) aminomethane pH 9.0. The immobilised pepsin was removed by centrifugation and the fragments were purified by anion exchange FPLC using a Mono QHR 5/5 column (Pharmacia LKB Biotechnology, Woerden, The Netherlands). Purity of both whole IgG and $F(ab')_2$ fragments was demonstrated by sodium dodecyl sulphate-polyacrylamide gel electrophoresis (SDS-PAGE).

*Cell lines*

The NIH: OVCAR-3 (OVCAR-3) human ovarian cancer cell line (Hamilton *et al.*, 1983) was kindly donated by Dr T.C. Hamilton (Fox Chase Cancer Center, Philadelphia, PA). WiDr is a human colon cancer cell line described by Noguchi *et al.* (1979). Colo 26 is a murine colon cancer cell line established from murine Colo 26 tumours described by Corbett *et al.* (1975). All cell lines were grown as a monolayer in Dulbecco's modified Eagle's medium (DMEM) obtained from Flow (Amsterdam, The Netherlands), supplemented with heat-inactivated 10% fetal calf serum (FCS).

*Tumour lines*

OVCAR-3 and WiDr xenografts were established from cells grown *in vitro* and injected s.c. in both flanks of female, 8–10 week-old NMRI/Cpb (nu/nu) mice (Harlan/Cpb, Zeist, The Netherlands). OVCAR-3 xenografts showed a poorly to moderately differentiated serous adenocarcinoma pattern, while that of WiDr was a poorly differentiated mucinous adenocarcinoma. Murine Colo 26 tumours were established in 7–8 week-old BALB/c mice (Harlan/Cpb, Zeist, The Netherlands) and histology revealed an undifferentiated pattern. The tumours were transferred by implanting fragments of solid tissue with a diameter of 2–3 mm s.c. through a small skin incision in subsequent recipients. Tumours were measured in three dimensions and the volume was expressed by the equation length × width × height × 0.5 in $mm^3$. The volume doubling times for OVCAR-3, WiDr and Colo 26 tumours were 10, 8 and 2½ days, respectively. Binding of MAbs and $F(ab')_2$ fragments to tumour cells was determined in an indirect immunoperoxidase assay as has been described earlier (Molthoff *et al.*, 1991a).

*Radiolabelling of antibodies and fragments*

Whole antibodies and fragments were labelled with either $^{125}I$ or $^{131}I$ by the one-vial iodogen method (Haisma *et al.*, 1986). Free iodine was removed by an anion exchange resin suspension in PBS containing 1% BSA (AG1-X8, Bio-Rad, Utrecht, The Netherlands). The percentage of radioactive iodine bound to the MAb was determined by trichloroacetic acid (TCA) precipitation and was always >95%. The specific activities of the iodinated MAbs ranged from 2 to 9 mCi/mg (Table II).

**Table II** Specific activity and immunoreactivity of MAbs and fragments

| *MAb* | IgG *SA** | IgG *IR*† | $F(ab')_2$ *SA* | $F(ab')_2$ *IR* |
|---|---|---|---|---|
| OC125 | 8.2 ± 0.2 | 70.4 ± 4.0 | 2.4 ± 1.0 | 83.8 ± 13.0 |
| OV-TL 3 | 9.4 ± 0.1 | 72.7 ± 7.0 | 2.6 ± 0.5 | 73.0 ± 6.3 |
| 139H2 | 4.9 ± 1.8 | 67.0 ± 5.4 | 1.9 ± 0.8 | 64.5 ± 7.6 |

*SA = specific activity in mCi $mg^{-1}$ ± s.d.; †IR = immunoreactivity in % ± s.d.

*Immunoreactivity and avidity*

After radiolabelling, the immunoreactive fraction was determined on OVCAR-3, WiDr or Colo 26 cells according to Lindmo *et al.* (1984). The immunoreactivities of the iodinated MAbs were in the range of 65 to 84% (Table II). The avidity of the specific MAbs was determined on OVCAR-3 cells by incubating a fixed amount of radiolabelled MAb mixed with unlabelled MAb over a concentration range of 1–150 µg $ml^{-1}$. The cell-bound radioactivity was measured in a gamma counter. The apparent affinity constant (avidity) was calculated from a Scatchard plot of specifically bound MAb versus bound over free MAb.

*Pharmacokinetics and biodistribution*

In mice, thyroid uptake of iodide was blocked by potassium iodide to the drinking water (0.1%) from 3 days before until the end of the study. Animals bearing OVCAR-3, WiDr or Colo 26 tumours (120 ± 51 $mm^3$) were injected with combinations of two radiolabelled MAbs (7–15 µCi per MAb), since all MAbs were directed against different antigenic determinants and the steric configuration did not hinder each other's binding. Mice were sacrificed at 1, 3, 6, 15, 24, 48, 72 and 168 h after injection. For each time point three mice were used. Blood was collected from mice under ether anaesthesia. Thereafter, normal tissues and tumours were dissected, rinsed in saline and dried to minimise blood residues. Blood and all tissues were weighed and the radioactivity was measured in a two-channel gamma counter with automatic correction for spillover of both radionuclides in the channels. To correct for radioactive decay, a standard solution of the injected material was prepared and counted simultaneously with the tissues at each day studied. The uptake of antibody was expressed as the percentage of injected dose per gram. The proportion of radioactivity associated with protein in serum was determined by precipitation with 10% TCA. Immune complex formation was analysed using gel filtration Superose-6 or Superose-12 FPLC chromatography.

*Radiation dose measurements and calculations*

The approximate radiation doses to various tissues were derived from the uptake data of the conjugate in each tissue assuming uniform distribution of the radionuclide within the organs. The absorbed dose was then calculated using the trapezoid integration method for the area under the curve (AUC) (Badger *et al.*, 1985). These doses were expressed in cGy by multiplying the integrated µCi h $g^{-1}$ by the g.cGy µ$Ci^{-1}$ $h^{-1}$ factor for $^{131}I$ published by the Medical Internal Radiation Dose committee (Dillman, 1969). The gamma-radiation dose has been neglected because of low absorbed fractions in the small organs of the mouse. The initial concentration of the radiolabelled MAb in each organ was assumed to be 0 $\mu$Ci $g^{-1}$.

### Results

### In vitro *characteristics of MAbs*

In an immunoperoxidase assay of cytospin preparations of OVCAR-3 cells, MAbs OC125, OV-TL 3 and 139H2 (whole antibody as well as fragments) were shown to strongly stain these cells, whereas MAbs 2C7 and A2C6 were negative (results not shown). None of the antibodies reacted with WiDr and Colo 26 cells.

Immunoperoxidase staining of tissue sections of OVCAR-3 xenografts with MAbs OC125 and 139H2 was intense and heterogeneously distributed. The binding of MAb 139H2 in the more differentiated areas of the tumour was mainly restricted to the apical cell membrane of the cells. Staining with MAb OV-TL 3 showed a more homogeneous pattern in OVCAR-3 tissue sections. No differences in staining were observed between MAbs as IgG or as $F(ab')_2$ fragments. MAbs 2C7 and A2C6 did not react with OVCAR-3 tumour tissue. WiDr tissue sections showed weak staining for MAb OV-TL 3, and were negative for the other MAbs.

A Scatchard analysis was performed with the radiolabelled MAbs on OVCAR-3 cells. The avidity, calculated as apparent affinity constants, for MAbs OC125, OV-TL 3 and 139H2 were $1 \times 10^9$ $M^{-1}$, $1 \times 10^9$ $M^{-1}$ and $1 \times 10^8$ $M^{-1}$, respectively (Table I). The number of antigenic sites per OVCAR-3 cell for these MAbs was in the range of $1 \times 10^6$ to $7 \times 10^6$ per cell.

### *Pharmacokinetics of MAbs and fragments*

OVCAR-3 bearing mice were injected with MAbs as IgG with either the combination of $^{131}$I-MAb OV-TL 3 plus $^{125}$I-MAb OC125 or with $^{131}$I-MAb 139H2 plus $^{125}$I-MAb 2C7. Since $F(ab')_2$ fragments of MAb 2C7 could not be made, tumour-bearing mice were injected with MAbs as $F(ab')_2$ fragments with the combination of $^{131}$I-MAb OV-TL 3 plus $^{125}$I-MAb OC125 or with $^{131}$I-MAb A2C6 plus $^{125}$I-MAb 139H2. Figure 1 shows the pharmacokinetics of the radiolabelled IgGs and $F(ab')_2$ fragments in the blood. The half-lives of MAbs OC125, OV-TL 3 and 139H2 were approximately 50 h for IgG and 6 h for $F(ab')_2$ fragments. A similar clearance pattern was observed for the control MAbs. The amount of free iodine in the serum, measured at each time point was $<10\%$. Serum radioactivity profiles corresponded to that of respectively IgG and $F(ab')_2$ fragments and no immune complexes were observed in the serum (not shown).

### *Biodistribution of MAbs and fragments*

Figure 2 shows the uptake of the radiolabelled MAbs as IgG and $F(ab')_2$ over a time period of 7 days in the OVCAR-3 tumours. The maximum % of the injected dose $g^{-1}$ in tumour tissue for MAbs OC125, OV-TL 3 and 139H2 as IgG was 8.5%, 17.7% and 11.1%, respectively. This level was reached 3 days after injection and the antibodies cleared slowly from the tumour with half-lifes of $>5$ days. In contrast, the maximum % injected dose $g^{-1}$ for the control MAb was 2.5% and no retention was observed. For the $F(ab')_2$ fragments, the maximum % of the injected dose $g^{-1}$ in tumour tissue for MAbs OC125, OV-TL 3 and 139H2 was 5.2%, 10.0% and 5.5%, respectively. This level was reached 6 to 15 h after injection. After that, no retention of the fragments in the tumour was observed. The respective $F(ab')_2$ fragments cleared from the tumour with half-lives of 13 h, 33 h and 30 h, respectively. $F(ab')_2$ fragments of MAb A2C6 reached a maximum of 1.9% of the injected dose $g^{-1}$ tumour without retention. Results for IgG and fragments from the biodistribution experiments in OVCAR-3 bearing mice are summarised in Table III.

**Figure 1** Blood clearance of radiolabelled MAbs as IgG and $F(ab')_2$ fragments in nude mice bearing OVCAR-3 xenografts. (●), $^{125}$I-OC125; (■), $^{131}$I-OV TL 3; (▲), $^{131}$I-139H2; (◆), $^{125}$I-2C7, s.d. $<16\%$.

**Figure 2** Tumour uptake of radiolabelled MAbs as IgG and $F(ab')_2$ fragments in nude mice bearing OVCAR-3 xenografts. (●), $^{125}$I-OC125; (■), $^{131}$I-OV TL 3; (▲), $^{131}$I-139H2; (◆), $^{125}$I-2C7, s.d. $<16\%$.

For IgG as well as for $F(ab')_2$ fragments of the three MAbs OC125, OV-TL 3 and 139H2, uptake in normal organs was much lower than in tumours. At 72 h after injection, liver uptake for the 3 IgGs was 1.5% of the injected dose $g^{-1}$. The amount of $F(ab')_2$ fragments in the liver measured 1.8% of the injected dose $g^{-1}$ at 15 h after injection. Other tissues showed an equal or lower uptake compared with that in the liver, except for the kidneys with respect to the $F(ab')_2$ fragments. Tumour to non-tumour ratios varied largely between IgG and $F(ab')_2$ fragments (Table IV). For the $F(ab')_2$ fragments of the three MAbs, the tumour to blood ratios were approximately eight times as high at 48 h as for IgG at 168 h. Also, tumour to normal organ ratios were more favourable for $F(ab')_2$ fragments as compared to IgG.

For analysis of tumour specificity, biodistribution of MAbs was examined in mice bearing WiDr colon cancer xenografts, grown from a control cell line, which did not express the relevant antigens. WiDr bearing mice were injected with MAbs as IgG in a combination of $^{131}$I-MAb OV-TL 3 plus $^{125}$I-MAb OC125 IgG or with $^{131}$I-MAb 139H2. Serum half-lives for MAbs OC125, OV-TL 3 and 139H2 were 44 h, 50 h and 60 h, respectively (Figure 3). No major differences were observed for the uptake in normal tissues between the three MAbs. Tumour uptake of MAbs OC125 and 139H2 was low and in the same order of magnitude as the negative control MAb 2C7 in OVCAR-3 xenografts. Surprisingly, tumour uptake of MAb OV-TL 3 was similar to that in the OVCAR-3 xenografts. The maximum level was 18.3% of the injected dose $g^{-1}$ in tumour tissue and was retained for $\geqslant 5$ days.

WiDr tissue sections were scored weakly positive for binding with MAb OV TL 3 in the immunoperoxidase assay while the WiDr cell line was negative. As biodistribution of MAb OV TL 3 in WiDr colon cancer xenografts was suggestive for specific uptake, similar experiments were carried out in the murine Colo 26 tumour model. After administration of a tracer dose of $^{131}$I-MAb OV-TL 3 plus $^{125}$I-MAb 139H2, mice were sacrificed at 6 h, 24 h and 72 h after injection. Results are depicted in Figure 4. No specific tumour uptake was observed and biodistribution in normal organs was similar for both antibodies.

*Dosimetry*

Absorbed radiation doses to the blood, tumours and normal tissues delivered by the radiolabelled MAbs were calculated from the data of the biodistribution experiments. Results for the various tumour lines and MAbs are summarised in Table V. The absorbed radiation dose in OVCAR-3 xenografts delivered by 10 $\mu$Ci radiolabelled MAbs OC125, OV-TL 3 and 139H2 as IgG was 38 cGy, 86 cGy and 39 cGy, respectively. For the respective $F(ab')_2$ fragments, the doses were 6 cGy, 22 cGy and 9 cGy. Doses to the blood exceeded those to the tumours, with the exception of MAb OV-TL 3 for both IgG as well as $F(ab')_2$ fragments. Absorbed radiation doses from radiolabelled MAbs OC125 and 139H2 to WiDr colon cancer xenografts were nearly half of the doses delivered to OVCAR-3 tumours.

### Discussion

The MAbs OC125, OV-TL 3 and 139H2 react with distinct antigenic determinants associated with ovarian carcinomas. MAbs OC125 and OV-TL 3 have been introduced in the clinic for radioimmunoscintigraphy while MAb 139H2 is still under preclinical investigation. We have shown that MAb 139H2 can specifically localise in human tumour xenografts. In the present study, human cell lines grown *in vitro* and as xenografts were used to compare the binding characteristics of MAb 139H2 with those of MAbs OC125 and OV-TL 3. Binding of the three MAbs was specific for tumours expressing the relevant antigen. Comparison of the MAbs as IgG and $F(ab')_2$ fragments in OVCAR-3 xenografts revealed a 4–6 fold lower absorbed radiation dose to tumours, but considerably higher tumour to blood ratios for the $F(ab')_2$

**Table III** Comparison of IgG and $F(ab')_2$ fragments in OVCAR-3 bearing mice

| *MAb* | *IgG* *Tumour** | *Blood* ($t\frac{1}{2}$)† | *F(ab')₂* *Tumour* | *Tumour* ($t\frac{1}{2}$) | *Blood* ($t\frac{1}{2}$) |
|---|---|---|---|---|---|
| OC125 | 8.5 ± 1.9 (72) | 52 | 5.2 ± 0.9 (15) | 13 | 6 |
| OV-TL 3 | 17.7 ± 3.5 (72) | 50 | 10.0 ± 1.5 (6–15) | 33 | 7 |
| 139H2 | 11.1 ± 1.9 (72) | 48 | 5.5 ± 0.7 (15) | 30 | 6 |
| control | 2.5 ± 0.8 | 50 | 1.9 ± 0.2 | | 6 |

*Maximum percentage of injected dose $g^{-1}$ ± s.d., between parentheses, time of measurement (h). †Half-life (h).

**Table IV** Tumour/non-tumour ratios after injection of a tracer dose of radiolabelled MAbs

| *MAb* | *Time** | *Blood* | *Liver* | *Spleen* | *Intestine* | *Muscle* | *Femur* |
|---|---|---|---|---|---|---|---|
| *IgG* | | | | | | | |
| OC125 | 72 | 0.9 | 5.3 | 7.1 | 8.1 | 14.2 | 7.7 |
| | 168 | 1.0 | 5.0 | 6.2 | 9.7 | 12.5 | 8.3 |
| OV-TL 3 | 72 | 2.1 | 9.8 | 11.8 | 14.5 | 29.5 | 13.6 |
| | 168 | 4.0 | 19.2 | 19.2 | 37.3 | 48.0 | 32.0 |
| 139H2 | 72 | 1.2 | 5.8 | 7.4 | 8.7 | 22.2 | 7.4 |
| | 168 | 2.3 | 11.5 | 11.5 | 22.1 | 33.7 | 20.0 |
| control | 72 | 0.3 | 1.2 | 2.0 | 2.3 | 6.2 | 2.3 |
| | 168 | 0.2 | 1.8 | 1.8 | 4.6 | 5.3 | 3.5 |
| *F(ab')₂* | | | | | | | |
| OC125 | 24 | 2.1 | 5.0 | 6.8 | 11.6 | 26.6 | 12.0 |
| | 48 | 8.0 | 11.3 | 16.1 | 10.7 | 26.5 | 7.5 |
| OV-TL 3 | 24 | 4.9 | 17.0 | 14.8 | 19.2 | 58.1 | 24.9 |
| | 48 | 37.8 | 98.2 | 98.2 | 76.4 | 163.7 | 164.0 |
| 139H2 | 24 | 2.2 | 6.4 | 6.9 | 11.1 | 27.1 | 13.1 |
| | 48 | 20.2 | 42.6 | 46.3 | 67.2 | 54.0 | 90.0 |
| control | 24 | 0.2 | 0.8 | 0.6 | 2.7 | 7.1 | 3.3 |
| | 48† | | | | | | |

*h after injection; †not determined.

**Figure 3** Blood clearance and tumour uptake of radiolabelled MAbs as IgG injected into nude mice bearing WiDr xenografts. (●), $^{125}$I-OC125; (■), $^{131}$I-OV-TL 3; (▲)$^{131}$I-139H2, s.d. < 19%.

**Figure 4** Biodistribution of radiolabelled MAbs OV-TL 3 (■) and 139H2 (□) injected into Balb/c mice bearing Colo 26 tumours. For blood, tumour and normal organs, first 2 bars represent 6 h; second 2 bars, 24 h and third 2 bars represent 48 h after injection, s.d. < 12%.

fragments. Although a similar number of antigenic determinants per cell was found in the human ovarian cancer cell line OVCAR-3, uptake in OVCAR-3 xenografts was most favourable for MAb OV-TL 3 as compared to that of MAbs OC125 and 139H2. This finding could not be ascribed to a difference in immunoglobulin isotype, to different affinities for the target antigen or to immune complex formation.

The biodistribution studies in human tumour xenografts indicated specific tumour localisation for all 3 MAbs. The higher accumulation for MAb OV-TL 3 as compared to that of MAb OC125 is in agreement with the results of Boerman *et al.* (1990) with the use of the same OVCAR-3 model. Mosely *et al.* (1988) observed a similar pattern with the F(ab')$_2$ fragments of MAbs OC125 and OV-TL 3 in an intraperitoneal tumour model. Although the WiDr cell line did not express the OA3 antigen, uptake in WiDr xenografts of radiolabelled MAb OV-TL 3 was as high as in the OVCAR-3 xenografts. A likely explanation may be the induction of the OA3 antigen expression in WiDr cells grown as xenografts, because no specific tumour localisation was observed in murine Colo 26 tumours. Radiolabelled MAb 139H2 demonstrated a maximum tumour uptake of 11.1% of the injected dose g$^{-1}$, in OVCAR-3 bearing mice. In our OVCAR-3 model, a ranking efficiency in tumour localisation would follow OV-TL 3 > 139H2 > OC125.

Circulating antigen can cause immune complex formation resulting in altered pharmacokinetics of the MAb and reduced uptake of antibody in tumours. Both MAbs OC125 and 139H2 react with antigens which are shed from tumour cells. These circulating antigens, CA125 and episialin, are being used for the monitoring of ovarian cancer and breast cancer patients, respectively (Kenemans *et al.*, 1988). To date, a variety of antibodies reactive with circulating antigens, such as MAbs HMFG1, HMFG2, OC125 and also the anti-carcinoembryonic antigen (CEA) MAbs, have been applied to many patients for successful detection of tumour lesions. In OVCAR-3 bearing nude mice, low levels of CA125 and episialin could be measured in the circulation (Molthoff *et al.*, 1991a). We were unable to demonstrate the formation of immune complexes or accelerated clearance of radiolabelled MAbs OC125 or 139H2 administered to OVCAR-3 bearing nude mice. Therefore, circulating antigens could not account for the differences in tumour uptake between the three MAbs.

Antigen localisation and accessibility have been shown to influence the tumour uptake of MAbs *in vivo* and can be attributed to immunological and non-immunological factors (reviewed by Jain *et al.*, 1987). In this respect, important immunological aspects are the number of antigenic determinants per cell, the cellular site of antigen expression, and the differentiation status of the cell and of the tumour. Expression of episialin in normal glandular epithelia and well-differentiated carcinomas is heterogeneous and detectable mostly at the apical cell membrane (Hilkens *et al.*, 1989). An increase in episialin determinants is often found in less differentiated carcinomas, where the glycoprotein is detected both intracellularly and on the entire cell surface. As a consequence, episialin in normal epithelia and well-differentiated tumours would be less accessible for MAb

**Table V** Dosimetry* of radiolabelled MAbs in tumour-bearing mice

| | *OVCAR-3* | | | *WiDr* | | |
|---|---|---|---|---|---|---|
| | *Tumour* | *Blood* | *Liver* | *Tumour* | *Blood* | *Liver* |
| OC125 IgG | 38 | 64 | 12 | 24 | 75 | 16 |
| OC125 F(ab')$_2$ | 6 | 10 | 3 | | | |
| OV-TL 3 IgG | 86 | 51 | 14 | 75 | 55 | 16 |
| OV-TL 3 F(ab')$_2$ | 22 | 10 | 3 | | | |
| 139H2 IgG | 39 | 66 | 13 | 21 | 67 | 15 |
| 139H2 F(ab')$_2$ | 9 | 8 | 2 | | | |
| control IgG | 10 | 35 | 10 | | | |
| control F(ab')$_2$ | 2 | 11 | 2 | | | |

*Absorbed radiation doses in cGy 10 $\mu$Ci$^{-1}$ injected dose calculated over 0–168 h.

139H2. The CA125 antigen is also expressed heterogeneously, but on the entire cell membrane and no correlation has been observed with the differentiation status of the tumour (Zurawski *et al.*, 1988). The OA3 antigen is known to be homogeneously distributed in tumour tissue and detectable on the cell membrane as well as in the cytoplasm (Poels *et al.*, 1986). In the OVCAR-3 model, the homogeneous OA3 distribution could be an explanation for the increased tumour uptake of MAb OV-TL 3.

Upon comparison of the binding characteristics of the three MAbs, MAb OV-TL 3 would be most favourable for clinical application. However, human tumour models may not always predict the behaviour of a MAb administered to patients. MAb OV-TL 3 was found to react with certain subpopulations of human leucocytes and their progenitors (unpublished data). Cross-reactivity with circulating cells in patients has been described by Dillman *et al.* (1984) for an anti-CEA MAb, which was associated with serious toxicity. In the immunoscintigraphic study using radiolabelled MAb OV-TL 3 in ovarian cancer patients (Buist *et al.*, 1992), the binding to white blood cells did not seem to interfere with the imaging results or to cause side effects. Nevertheless, significant binding to non-target cells may pose a major limitation with respect to repeated administration or higher doses of MAbs into patients.

High tumour to normal tissue ratios are considered to be of advantage for the application of radiolabelled MAbs for the detection of tumour lesions in cancer patients. In our study, we observed higher tumour to non-tumour ratios after administration of radiolabelled $F(ab')_2$ fragments compared with IgG. Other investigators demonstrated improved immunoscintigraphic detection of experimental tumours using radiolabelled fragments (Buchegger *et al.*, 1983; Hansson *et al.*, 1988; Gerretsen *et al.*, 1991). Thus far, clinical data from radioimmunodetection of cancer confirmed an increase in sensitivity for $F(ab')_2$ fragments compared with intact IgG (Lamki *et al.*, 1990).

For effective radioimmunotherapy, high radiation doses absorbed in tumour tissue would be necessary. In the OVCAR-3 model, we found iodinated MAbs as IgG to deliver four to six times more radiation to tumours than the $F(ab')_2$ fragments. Earlier, we have demonstrated that high doses of $^{131}$I-labelled MAb 139H2 as IgG can induce complete tumour regressions of particular human ovarian cancer xenografts (Molthoff *et al.*, 1992). Buchegger *et al.* (1990) have suggested that repeated injections of high doses of $^{131}$I-labelled anti-CEA $F(ab')_2$ result in superior therapeutic effects compared with equitoxic doses of $^{131}$I-labelled IgG. In this respect, $F(ab')_2$ fragments may have an advantage because of the higher tumour to normal tissue ratios and the lower bone marrow toxicity from faster blood clearance. Moreover, smaller fragments of MAbs like $F(ab')_2$ or Fab' show a reduced immunogenicity compared with IgG. We consider the lower absolute uptake in tumour tissue and the short retention time as disadvantages for the use of $F(ab')_2$ fragments for treatment. Future studies should focus on the precise role of IgG *versus* $F(ab')_2$ in radioimmunotherapy.

From the three antibodies studied, MAb OV-TL 3 shows the most favourable tumour uptake, but the cross-reactivity with certain white blood cells may hamper its therapeutic use. Of interest, the investigational MAb 139H2 has binding characteristics which appear slightly better than MAb OC-125. MAb 139H2 is known to have a broad reactivity pattern with ovarian adenocarcinomas. Therefore, MAb 139H2 should be evaluated for its usefulness in the diagnosis of ovarian cancer patients.

This work was supported by the Dutch Cancer Society. We thank Dr H.J. Haisma for critically reading the manuscript.

### References

BADGER, C.C., KROHN, K.A., PETERSON, A.V., SCHULMAN, H. & BERNSTEIN, I.D. (1985). Experimental radiotherapy of murine lymphoma with $^{131}$I-labeled anti-Thy 1.1 monoclonal antibody. *Cancer Res.*, **45**, 1536.

BARNEVELD, R.A., TEGELAERS, F.P.W., GINNS, E.I. & 7 others (1983). Monoclonal antibodies against human β-glucocerebrosidase. *Eur. J. Biochem.*, **134**, 585.

BAST, R.C., FEENEY, M., LAZARUS, H., NADLER, L.M., COLVIN, R.B. & KNAPP, R.C. (1981). Reactivity of a monoclonal antibody with human ovarian carcinoma. *J. Clin. Invest.*, **68**, 1331.

BOERMAN, O., MASSUGER, L., MAKKINK, K., THOMAS, Ch., KENEMANS, P. & POELS, L. (1990). Comparative in vitro binding characteristics and biodistribution in tumor-bearing athymic mice of anti-ovarian carcinoma monoclonal antibodies. *Anticancer Res.*, **10**, 1289.

BUCHEGGER, F., HASKELL, C.M., SCHREYER, M. & 4 others (1983). Radiolabeled fragments of monoclonal antibodies against carcinoembryonic antigen of localization of human colon carcinoma grafted into nude mice. *J. Exp. Med.*, **158**, 413.

BUCHEGGER, F., PELEGRIN, A., DELALOYE, B., BISCHOF-DELALOYE, A. & MACHA, J.P. (1990). Iodine-131-labeled MAb $F(ab')_2$ fragments are more efficient and less toxic than intact anti-CEA antibodies in radioimmunotherapy of large human colon carcinoma grafted in nude mice. *J. Nucl. Med.*, **31**, 1035.

BUIST, M.R., KENEMANS, P., VERMORKEN, J.B. & 8 others (1992). Radioimmunotargeting in ovarian carcinoma patients with Indium-111 labeled monoclonal antibody OV-TL 3 $F(ab')_2$: pharmacokinetics, tissue distribution, and tumor imaging. *Int. J. Gynec. Cancer* (in press).

CORBETT, T.H., GRISWOLD, D.P., ROBERTS, B.J., PECKHAM, J.C. & SCHABEL, F.M. (1975). Tumor induction relationships in development of transplantable cancers of the colon in mice for chemotherapy assays, with a note on carcinogen structure. *Cancer Res.*, **35**, 2434.

DILLMAN, L.T. (1969). *Radionuclide decay schemes and nuclear parameters for use in radiation-dose estimation.* MIRD Pamphlet 4. The Society of Nuclear Medicine: New York.

DILLMAN, R.O., BEAUREGARD, J.C., SOBOL, R.E. & 4 others (1984). Lack of radioimmunodetection and complications associated with monoclonal anticarcinoembryonic antigen antibody cross-reactivity with an antigen on circulating cells. *Cancer Res.*, **44**, 2213.

GERRETSEN, M., QUAK, J.J., SUH, J.S. & 4 others (1991). Superior localisation and imaging of radiolabelled monoclonal antibody E48 $F(ab')_2$ fragment in xenografts of human squamous cell carcinoma of the head and neck and of the vulva as compared to monoclonal antibody E 48 IgG. *Br. J. Cancer*, **63**, 37.

HAISMA, H.J., HILGERS, J. & ZURAWSKI, V.R. (1986). Iodination of monoclonal antibodies for diagnosis and radiotherapy using a convenient one vial method. *J. Nucl. Med.*, **27**, 1890.

HAMILTON , T.C., YOUNG, R.C., MCKOY, W.M. & 7 others (1983). Characterization of a human ovarian cancer carcinoma cell line (NIH: OVCAR-3) with androgen and estrogen receptors. *Cancer Res.*, **43**, 5379.

HANSSON, Y., PAULIE, S., BEN-AISSA, H., RUDBERG, U., KARLSSON, A. & PERLMANN, P. (1988). Radioimmunolocalisation of bladder tumors xenotransplanted in nude mice. *Anticancer Res.*, **8**, 435.

HILKENS, J., BUYS, F. & LIGTENBERG, M. (1989). Complexity of MAM-6, an epithelial sialomucin, associated with carcinomas. *Cancer Res.*, **49**, 786.

JAIN, R.K. (1987). Transport of molecules in the tumor interstitium: a review. *Cancer Res.*, **47**, 3039.

KENEMANS, P., BAST, R.C., YEDEMA, C.A., PRICE, M.R. & HILGERS, J. (1988). CA125 and polymorphic epithelial mucin as serum tumor markers. *Cancer Rev.*, **11–12**, 119.

LAMKI, L.M., PATT, Y.Z., ROSENBLUM, M.G. & 5 others (1990). Metastatic colorectal cancer: radioimmunoscintigraphy with a stabilized In-111-labeled $F(ab')_2$ fragment of an anti-CEA monoclonal antibody. *Radiology*, **174**, 147.

LINDMO, T., BOVEN, E., CUTTITTA, F., FEDORKO, J. & BUNN, P.A. (1984). Determination of the immunoreactive fraction of radiolabeled monoclonal antibodies by linear extrapolation to binding at infinite antigen excess. *J. Immunol. Methods*, **72**, 77.

MASSUGER, L.F.A.G., KENEMANS, P., CLAESSENS, R.A.M.J. & 6 others (1990). Immunoscintigraphy of ovarian cancer with indium-111-labeled OV-TL 3 F(ab')$_2$ monoclonal antibody. *J. Nucl. Med.*, **31**, 1802.

MOLTHOFF, C.F.M., CALAME, J.J., PINEDO, H.M. & BOVEN, E. (1991a). Human ovarian cancer xenografts in nude mice: characterization and analysis of antigen expression. *Int. J. Cancer*, **47**, 72.

MOLTHOFF, C.F.M., PINEDO, H.M., SCHLÜPER, H.M.M. & BOVEN, E. (1991b). Pharmacokinetics and biodistribution of a new antiepisialin monoclonal antibody 139H2 in ovarian cancer bearing nude mice. *Cancer Immunol. Immunother.*, **34**, 191.

MOLTHOFF, C.F.M., PINEDO, H.M., SCHLÜPER, H.M.M. & BOVEN, E. (1992). Influence of close and schedule on the therapeutic efficacy of $^{131}$I-labelled monoclonal antibody 13gH2 in a human ovarian cancer xenograft model. *Int. J. Cancer* (in press).

MOSELY, K., BATTAILLE, A., KNAPP, R.C. & HAISMA, H.J. (1988). Localization of radiolabelled F(ab')$_2$ fragments of monoclonal antibodies in nude mice bearing intraperitoneally growing human ovarian cancer xenografts. *Int. J. Cancer*, **42**, 368.

NOGUCHI, P., WALLACE, R. & JOHNSON, J. (1979). Characterization of WiDr: a human colon carcinoma cell line. *In Vitro*, **15**, 401.

POELS, L.G., PETERS, D., VAN MEGEN, Y. & 7 others (1986). Monoclonal antibody against human ovarian tumor-associated antigens. *JNCI*, **76**, 781.

TAYLOR-PAPADIMITRIOU, J., PETERSON, J., ARKLIE, J., BUOCHELL, J., CERARI, R.L. & BODMER, W. (1981). Monoclonal antibodies to epithelium – specific components of the human milk fat globule membrane: production and reaction with cells in culture. *Int. J. Cancer*, **28**, 17.

RAINSBURY, R.M., WESTWOOD, J.H., COOMBES, R.C. & 5 others (1983). Location of metastatic breast carcinoma by a monoclonal antibody chelate labelled with indium-111. *Lancet*, **ii**, 934.

ZURAWSKI, V.R. & MATTIS, J.A. (1983). Detection of hepatitis B surface antigen using monoclonal antibodies. In *Clinical Laboratory Assays: New Techniques and Future Directions*, Nakamura, T. (ed.), p. 361. Mason Publishing Company: New York.

ZURAWSKI, V.R., DAVIS, H.M., FINKLER, N.J., HARRISON, C.L., BAST, R.C. & KNAPP, R.C. (1988). Tissue distribution and characteristics of the CA125 antigen. *Cancer Rev.*, **11–12**, 102.